# Distribution of the invasive bryozoan *Schizoporella japonica* in Great Britain and Ireland and a review of its European distribution

**DOI:** 10.1007/s10530-017-1440-2

**Published:** 2017-04-28

**Authors:** J. Loxton, C. A. Wood, J. D. D. Bishop, J. S. Porter, M. Spencer Jones, C. R. Nall

**Affiliations:** 1 The Environmental Research Institute, North Highland College, The University of the Highlands and Islands, Ormlie Rd, Thurso, KW14 7EE Scotland, UK; 2 The Laboratory, The Marine Biological Association of the UK, Citadel Hill, Plymouth, PL1 2PB UK; 3 International Centre for Island Technology, Heriot Watt University Orkney Campus, The Old Academy, Back Road, Stromness, Orkney, KW16 3AW Scotland, UK; 4 Department of Life Sciences, Natural History Museum, London, SW7 5BD UK

**Keywords:** *Schizoporella japonica*, Bryozoa, Europe, Biological invasion, Non-indigenous species

## Abstract

The bryozoan *Schizoporella japonica* Ortmann (1890) was first recorded in European waters in 2010 and has since been reported from further locations in Great Britain (GB) and Norway. This paper provides a new earliest European record for the species from 2009, a first record from Ireland and presence and absence records from a total of 231 marinas and harbours across GB, Ireland, the Isle of Man, France and Portugal. This species is typically associated with human activity, including commercial and recreational vessels, aquaculture equipment, and both wave and tidal energy devices. It has also been observed in the natural environment, fouling rocks and boulders. The species has an extensive but widely discontinuous distribution in GB and Ireland. Although found frequently in marinas and harbours in Scotland, it inhabits only a few sites in England, Wales and Ireland, interspersed with wide gaps that are well documented as genuine absences. This appears to be a rare example of a southward-spreading invasion in GB and Ireland. The species has been reported from the Isle of Man and Norway but has not been found in France or Portugal. In the future we expect *S. japonica* to spread into suitable sections of the English, Welsh and Irish coasts, and further within Europe. The species’ capability for long-distance saltatory spread and potential for negative impact on native ecosystems and economic activity suggests that *S. japonica* should now be considered invasive in GB and Ireland. As such, it is recommended that biosecurity procedures alongside effective surveillance and monitoring should be prioritised for regions outside the species’ current distribution.

**Electronic supplementary material:**

The online version of this article (doi:10.1007/s10530-017-1440-2) contains supplementary material, which is available to authorized users.

## Background

For millennia, the natural geographical and biological barriers in the oceans have provided levels of isolation essential for species and distinct ecosystems to evolve. However, since the advent of ship travel people have been inadvertently or deliberately carrying marine organisms into new habitats, where they can become established as non-native or invasive species (Keller et al. 2011). Globalisation and growth in trade and tourism provide more opportunities than ever before for species to be spread. An increasing list of marine vectors is associated with commercial shipping, aquaculture, fisheries, mineral extraction, recreational boating, marine sports and diving, the aquarium and live-bait trades, floating debris and canals linking water bodies. In the marine environment, bryozoans, sea-squirts, sponges, mussels, barnacles and other sessile, fouling organisms constitute the majority of non-native species, as they are able to attach to boat hulls, ballast tanks, solid ballast and aquaculture imports.

The introduction of non-native bryozoan species has had significant and wide-ranging impacts worldwide. *Schizoporella errata* is a vigorously invasive species which is now widespread throughout the world’s warm temperate to subtropical waters (Hayward and McKinney 2002). It is a strong competitor for space and is known to inhibit the growth of adjacent species. It is considered to be an ‘ecosystem engineer’ due to its ability to form massive encrustations, overgrow other organisms and modify habitat characteristics. Since the discovery of *Tricellaria inopinata* in the Lagoon of Venice in 1982, this arborescent species has spread rapidly throughout European waters at a rate of 190 km yr^−1^, probably facilitated by its ability to colonise a wide range of biotic and abiotic substrates (Cook et al. 2013). *Tricellaria inopinata* was found on over 85% of yacht hulls in both Plymouth, UK and Brittany, France (JDDB and ALE Yunnie, unpubl. obs.) and has been listed amongst problematic species in relation to fuel consumption by commercial ships. *Tricellaria inopinata* has also had a profound influence on the community of small sessile invertebrates growing in the Lagoon of Venice, including overgrowth of mussels and other calcareous organisms and apparent displacement of native bryozoans. *Membranipora membranacea*, an abundant European native species, is now invasive in North America where it is negatively impacting kelp beds and other canopy-forming seaweeds, with a significant effect on macroalgal reproduction and dynamics and an increased susceptibility to storm damage. In the aquaculture industry, bryozoans have been also shown to impact the cultivation efficiency of kelp, and shellfish.

The cheilostomatous bryozoan *Schizoporella japonica* was first brought to general notice as a non-native species in British marine waters in 2014, after it had been observed in Wales (2010) and Scotland (2011) (Ryland et al. 2014); the species has since been reported in further locations in GB (Collin et al. 2015; Nall et al. 2015 and Bishop et al. 2015b). *S. japonica* was first described by Ortmann in 1890 from Japan under the name *Schizoporella unicornis* var. *japonica*. Its native distribution is ascribed to the North-West Pacific from China to Japan (Dick et al. 2005). *Schizoporella japonica* is an encrusting species which forms bright orange-red, calcified crusts with distinctive foliose lobes in well-developed colonies (Ryland et al. 2014), hence the common name of the Orange Ripple Bryozoan. Within its natural range, *S. japonica* is predominantly found intertidally on rocks, boulders and shellfish (Dick et al. 2005), although worldwide it has diversified onto man-made hard substrates such as pilings, hulls and pontoons.

Each colony begins with a single, sexually produced, primary zooid, which then buds asexually to form unilaminar or bilaminar sheets. *Schizoporella japonica* is hermaphroditic and bright orange embryos are brooded in external brood chambers (ovicells) (Dick et al. 2005). *Schizoporella japonica* is unusual in that it is one of only a handful of bryozoan species worldwide which exhibit multiple ovicells on a single zooid. This trait has been observed extensively in British specimens (Ryland et al. 2014) and also recorded in North American and Japanese material (Powell 1970). It is not known if this feature is an aberration caused by pollution, as was originally proposed by Powell (1970), or a natural modification of the prevalent reproductive pattern. Larvae are ciliated and non-feeding, and have a preference to attach and metamorphose within hours following release. *S. japonica* is tolerant of salinities from 15 to 36 (Powell 1970; Loxton 2014) and temperatures from 4 to 30 °C (Loxton 2014; Taylor and Tan 2015; NOAA 2016). Actively reproducing colonies have been observed in British midwinter (Ryland et al. 2014; Loxton 2014) and larval settlement has been observed year-round in Charleston, Oregon where temperatures are between 7 and 15 °C (Treibergs 2012).

*Schizoporella japonica* is thought to have begun its journey into non-native waters as an encrusting hitchhiker on live Pacific oysters, *Magallana gigas*, which were exported extensively in the early twentieth century from Japan to the west coast of North America for aquaculture (Powell 1970; Ryland et al. 2014). The first documentation of the bryozoan in North America was in 1938 from Los Angeles and it has since made its way northwards as far as Alaska (Dick et al. 2005). More recent reports for *S. japonica* suggest that it is well established along the West coast of Canada and the USA (Treibergs 2012; Ashton et al. 2014), where it is reported to be found on both man-made and natural intertidal substrates. *S. japonica* was first observed in Europe in 2010 when samples were identified from Holyhead in Wales and shortly afterwards from Orkney in Scotland (Ryland et al. 2014). It has since been reported from further locations in Scotland (Collin et al. 2015; Nall et al. 2015) and England (Bishop et al. 2015b). In 2014, *S. japonica* was found in three marinas in Norway (Porter et al. 2015). In Europe, it has been observed predominantly on man-made structures with the exception of observations of its occurrence on rocks and boulders in areas close to harbours: Lerwick, Shetland (Collin et al. 2015) and Stromness, Orkney (JL and CRN, unpubl. obs.). Its worldwide distribution also includes Langkawi, Malaysia, where reproductive colonies were observed by Taylor and Tan (2015), and may also include Australia where *S. unicornis* (possibly *S. japonica*) was reported in 1975 following imports of Pacific oysters (Dick et al. 2005). The exact global range of *S. japonica* is currently unclear, as it has commonly been misidentified as *S. unicornis* or *S. errata* (Dick et al. 2005; Treibergs 2012; Ryland et al. 2014).

The potential for commercial and ecological damage caused by non-native species, such as *S. japonica*, and the subsequent need for effective surveillance and monitoring, are becoming an increasing focus for industry, governments, regulators and conservationists (Keller et al. 2011; Collin et al. 2015). Since its first recording in Europe 5 years ago *S. japonica* has been observed fouling a variety of commercial assets including shellfish and finfish aquaculture equipment (Collin et al. 2015), ferries and commercial vessels (Ryland et al. 2014), recreational vessels, marina pontoons and fenders (Collin et al. 2015; Bishop et al. 2015b) and both wave (Nall 2015) and tidal (Ryland et al. 2014) energy extraction devices. Additionally the first observation of *S. japonica* in the natural environment was noted in Shetland in 2015 (Collin et al. 2015), which brings the threat of ecological damage to native communities to the fore.

*Schizoporella japonica* is included in the priority list compiled in 2015 of 24 marine non-indigenous species to be monitored in the UK for Descriptor 2 of Good Environmental Status (GES) under the Marine Strategy Framework Directive (EU directive 2008/56/EC) (MSFD). Inclusion was based on the species’ potential impact as estimated in a range of official risk assessment exercises. If this directive remains in force, the species’ distribution around the UK will be monitored over future years and contribute to an assessment of the UK’s adherence to ensuring GES. The aim of the present study was to collate information from literature and recent surveys in GB, Ireland, Portugal and NW France to assemble the best available picture of the current distribution of *S. japonica* in Europe. We also discuss vectors likely to have been involved in the species’ movement and its potential for further spread.

## Methods

Surveys for *S. japonica* were conducted by the authors between 2011 and 2016 at a total of 216 marinas and harbours across GB, Ireland, France and Portugal, of which 94 have previously been reported in scientific papers or reports, and 122 are first reported here. See Supplementary information for details of the sites, references and survey methods used. Rapid assessment surveys (RASs) with *S. japonica* as one of the target species were made as described by Bishop et al. (2015b). Some English sites names are coded to preserve anonymity at the request of the site owners as a condition of access. The authors’ personal collections of fouling bryozoans were also revisited to check for any unidentified samples that may have contained *S. japonica*, which produced one additional record. In rapid assessment surveys, and in visits specifically to search for *S. japonica,* submerged structures within each marina/harbour were inspected from the pontoons and included pontoon floats, hanging chains and ropes, harbour walls, vessel hulls and buoys/fenders. Most of the surfaces accessed were thus relatively shallow, to a depth of approximately 0.5 m. At all sites, when potential colonies were found, a sample was collected and preserved for later identification under an optical microscope.

Identification of all samples was based on Dick et al. (2005), Tompsett et al. (2009) and Ryland et al. (2014) and conducted by experienced bryozoan taxonomists. Representative samples were examined using optical microscopes in all cases, and using scanning electron microscopes where further verification was required. Previous publications have highlighted the potential for confusion between *Schizoporella japonica* and the UK native *S. unicornis* and the non-native species *S. pseudoerrata* and *S. errata* (Tompsett et al. 2009; Ryland et al. 2014). In this context of possible confusion, the authors would point out that in GB and Norway the native *S. unicornis* is hardly ever seen as a fouling organism (Ryland et al. 2014), but it is common and well-known on natural substrates on the lower shore or sublittorally and all authors are familiar with identifying it under a microscope. *S. errata*, despite its inclusion in the Linnean Synopses and other species lists of British *Schizoporella* (Hayward and Ryland 1995), has never been found as an established population in the UK. *S. pseudoerrata* has only been recorded from the US Pacific coast to date (Ryland et al. 2014). Nethertheless precautions have been taken to ensure no misidentification occurred in this study. Characters checked include: the shape and placing of oral avicularia; the occasional presence of large frontal avicularia and orifice closure plates; the occurrence of multiple ovicells per zooid; the proximal edge of the orificial sinus often being slightly or markedly straightened; distinctive condyles; and entire frontal surface of the ovicell porous, generally with radiating ridges between pores. More information on these distinguishing characters can be found in Dick et al. (2005) and Ryland et al. (2014). Published scientific papers and reports other than the authors’ own were also reviewed for sightings throughout Europe, and provided a further 4 presence and 11 absence records for the species. See Supplementary material for full details of these sources. Absences were only included where *S. japonica* had specifically been on the list of targeted species in surveys, but had not been found. All additional presence records were checked/validated to ensure the identification had been appropriately verified.

## Results

### Distribution in Great Britain

**Fig. 1:**
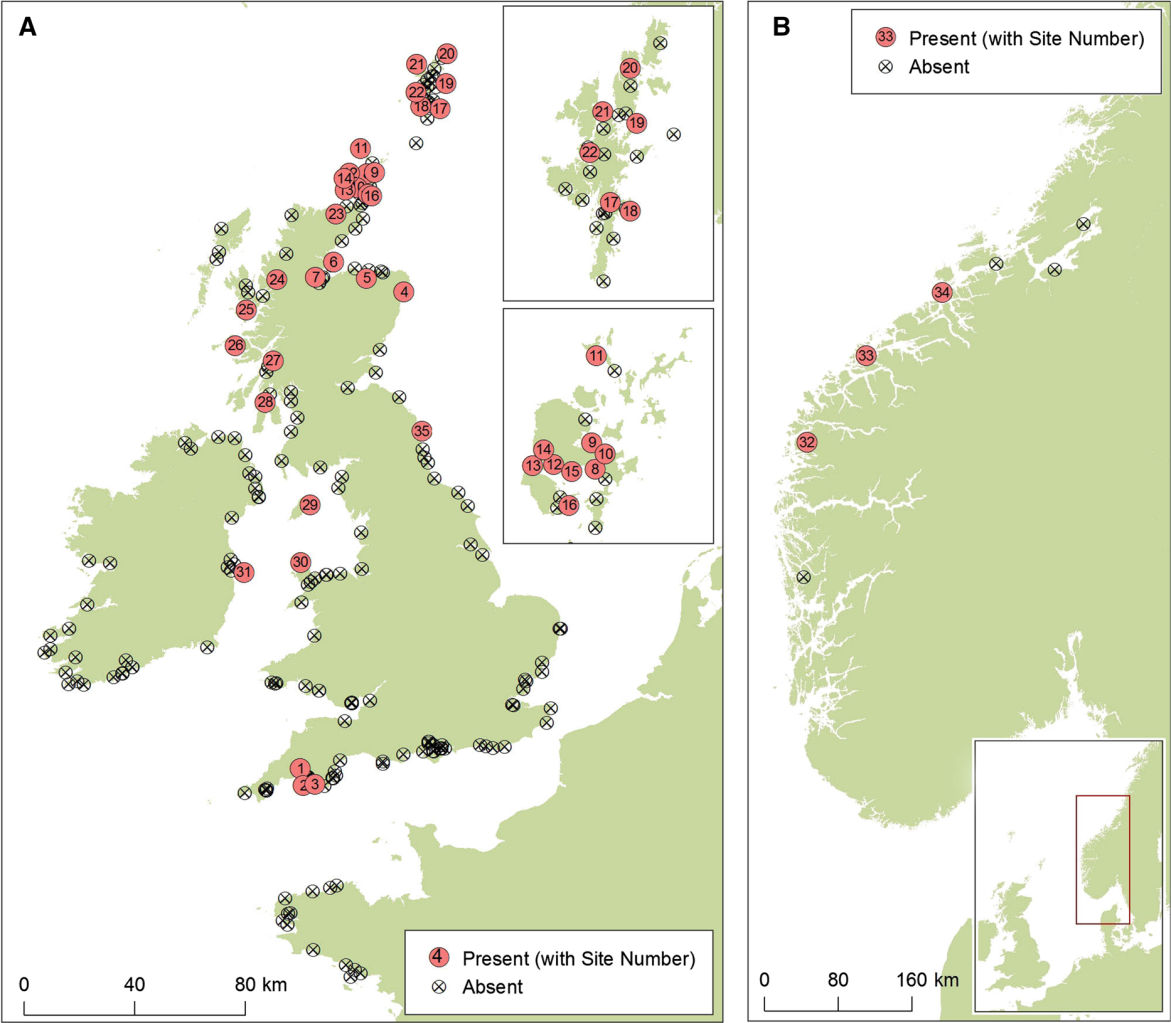
Maps of *Schizoporella japonica* presence and absence in Great Britain, Ireland, the Isle of Man and France (**a**) and Norway (**b**). *Insets* in **a** show Orkney (*bottom*) and Shetland (*top*). *Filled circles* indicate presence records with site numbers as designated in Table 1. *Hollow circles* with crosses indicate absence records. See the Supplementary information for full details of presence and absence records

*Schizoporella japonica* was found to have an extensive but widely discontinuous distribution in GB (Fig. 1; Table 1). In 2010 *S. japonica* was found in Holyhead, and 6 years later, despite it being actively looked for at 15 sites, this remains its only known locality in Wales. Until recently *S. japonica* had not been recorded in England beyond a small area in Plymouth on the south coast. This paper adds a new very recent record for the species in S. Northumberland, NE England, although the distribution in England remains extremely discontinuous. In Scotland *S. japonica* displays a more continuous distribution as it has successfully established populations in marinas on both the east and west coasts of Scotland and in both Orkney and Shetland. In the north of Scotland there was also evidence for the spread of *S. japonica* as it had been observed at a number of sites in 2014 (Scapa, Houton and Hoy Lyness) where it had previously been recorded absent from RAS surveys in 2012 (Nall et al. 2015).

**Table 1:** The presence records of *Schizoporella japonica* at 35 sites in Europe, out of the 231 sites surveyed

	Location	Lat.	Long.	First observed	Surveyor(s)	References
1	Plymouth 2	50.3667	−4.1555	04/10/2013	JDDB/CAW/AY	Wood et al. (2015)
2	Plymouth 3	50.3634	−4.1522	28/11/2012	JDDB/CAW/AY	Bishop et al. (2015b)
3	Plymouth 5^a^	50.3652	−4.1309	05/11/2009	JDDB	
4	Peterhead Bay Marina	57.4957	−1.7912	13/11/2012	JL/JM/JSP	
5	Portnockie Harbour	57.7046	−2.8621	13/11/2012	JL/JM/JSP	
6	Cromarty	57.6830	−4.0379	30/08/2012	CRN	Nall et al. (2015)
7	Invergordon	57.6858	−4.1680	31/08/2012	CRN	Nall et al. (2015)
8	Scapa	58.9565	−2.9713	17/07/2014	Orkney MEU	DASSH
9	Kirkwall Marina	58.9872	−2.9582	01/09/2011	JL/JSP	
10	Kirkwall Hatson	58.9999	−2.9747	08/08/2012	CRN	Nall et al. (2015)
11	Westray Pierowall	59.3233	−2.975	09/08/2012	CRN	Nall et al. (2015)
12	Stromness Marina	58.9638	−3.2957	12/02/2011	JL	
13	Stromness intertidal^c^	58.9601	−3.2996	13/08/2014	JL/CRN	
14	Stromness Polestar	58.9590	−3.2993	07/08/2012	CRN	Nall et al. (2015)
15	Houton	58.9172	−3.1851	16/07/2014	Orkney MEU	DASSH
16	Hoy Lyness^b^	58.8367	−3.19	20/04/2014	CRN	
17	Lerwick: Victoria Pier^c^	60.1538	−1.1412	26/06/2012	JSP	Collin et al. (2015)
18	Lerwick: Gremista Marina	60.1691	−1.1621	01/09/2012	JL/JSP	
19	Burravoe	60.4976	−1.0426	01/09/2012	JL/JSP	
20	Cullivoe	60.6986	−0.9962	14/08/2014	JSP	
21	Collafirth	60.4018	−1.2149	13/06/2013	JSP	Collin et al. (2015)
22	Brae	60.3916	−1.3654	24/05/2013	JSP	Collin et al. (2015)
23	Scrabster Harbour	58.6110	−3.5456	21/08/2012	CRN	Nall et al. (2015)
24	Kishorn Port	57.3931	−5.6389	10/11/2015	JL	
25	Mallaig Marina	57.0078	−5.8261	27/08/2014	JL	
26	Tobermory Marina	56.6200	−6.0669	01/08/2013	JL	
27	Croabh Yacht Haven	56.2107	−5.5576	23/03/2012	CRN	Nall et al. (2015)
28	Portavadie	55.8748	−5.3131	26/10/2012	JL/JSP	
29	Douglas, Isle of Man	54.1469	−4.4726	04/04/2014	EJC	Pers comms. Liz Cook
30	Holyhead Marina	53.3204	−4.6434	01/05/2011		Ryland et al. (2014), Sambrook et al. (2014)
31	Greystones Marina, Ireland	53.1523	−6.0663	26/10/2015	JL	
32	Florø	61.6012	5.0356	30/06/2014	JSP/MSJ	Porter et al. (2015)
33	Ålesund, Brunholmkaia	62.4737	6.1554	01/07/2014	JSP/MSJ	Porter et al. (2015)
34	Kristiansund, Vaagakaia	63.1135	7.7328	02/07/2014	JSP/MSJ	Porter et al. (2015)
35	S. Northumberland	55.1193	−1.4954	21/07/2016	JDDB/CAW/CRN	

For full details of references see Supplementary material

^a^Indicates found to be present on this date only, but not subsequent inspections

^b^Indicates found on the Pelamis wave device

^c^Indicates found on natural substrate in the intertidal rocky shoreline

**Fig. 2:**
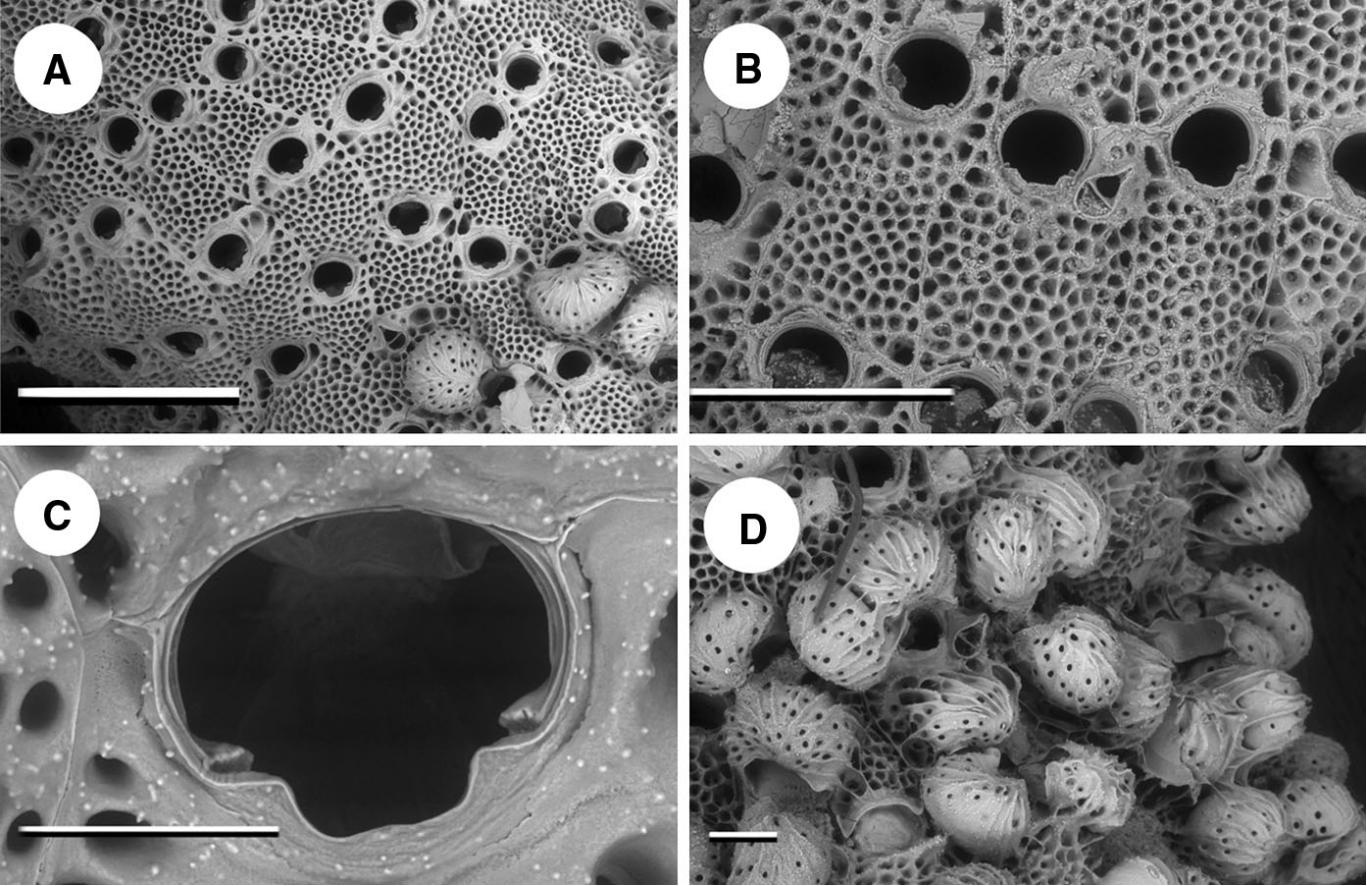
Scanning electron microscope images of bleached specimens from Plymouth 5 collected in November 2009. **a** Scale = 1 mm. **b** Showing oral avicularium, scale = 500 µm. **c** Orifice with distinctive shape and condyles, scale = 100 µm. **d** Zooids with multiple ovicells, scale = 200 µm

**Fig. 3:**
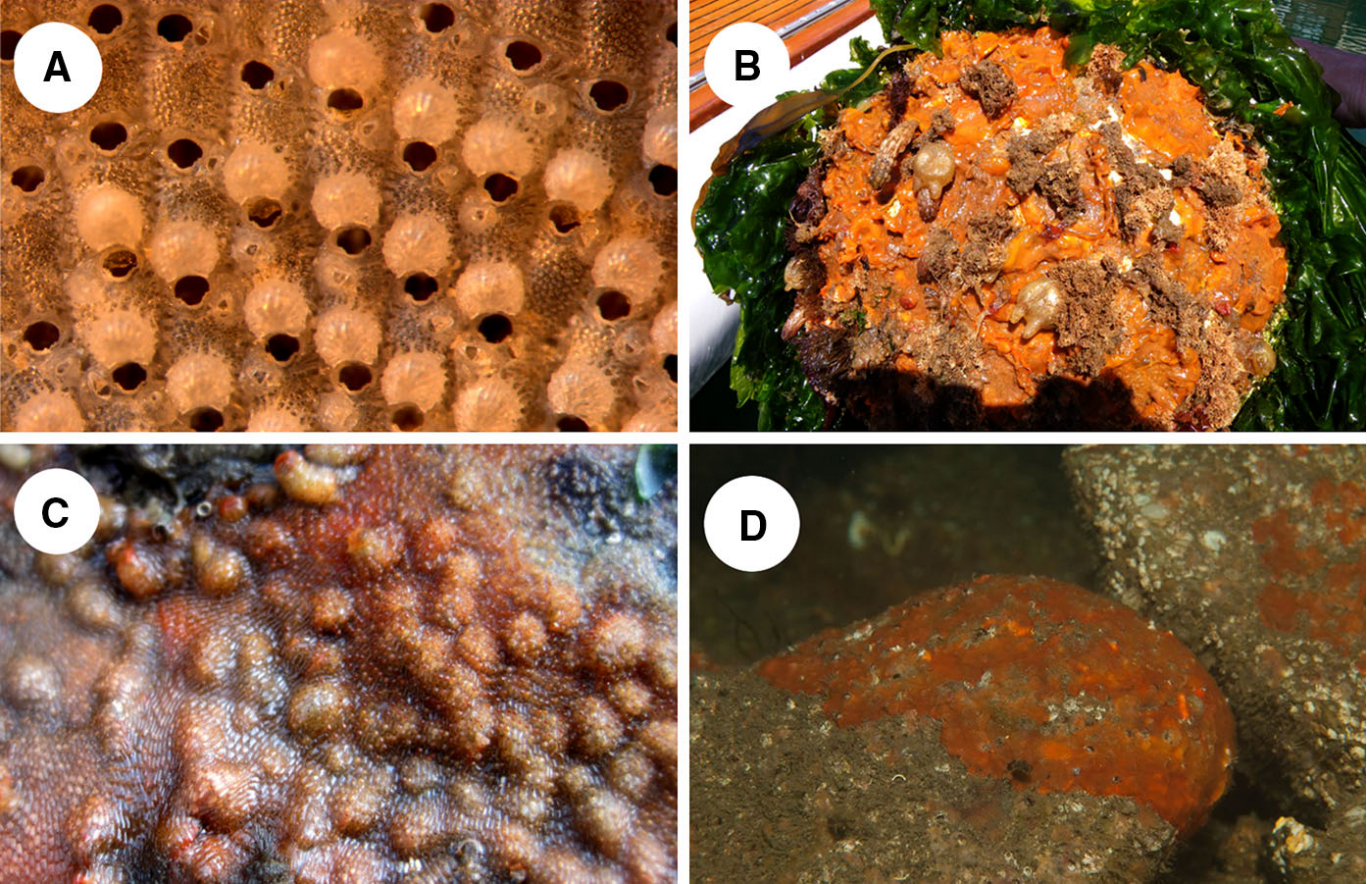
Images of *Schizoporella japonica*. **a** Light microscopy image of *S. japonica* from Greystones Marina, Ireland. **b** *S. japonica* fouling a buoy (Plymouth 3, Table 1). **c** *S. japonica* colonies on an intertidal rock in Stromness, Orkney. **d** Underwater photograph of *S. japonica* on boulders in Lerwick, Shetland

A sample was revisited from JDDB’s collection which had been collected from a marina in Plymouth (Plymouth 5 in Table 1) in 2009 and simply labelled “*Schizoporella* sp. orange”. On re-examination this was identified as *S. japonica*, imaged using SEM (Fig. 2), and has been registered in the Natural History Museum collection under number NHMUK 2015.4.20.1. This sample now supersedes the 2010 Holyhead record in Ryland et al. (2014) as the first record of *S. japonica* in Europe. Subsequent visits to Plymouth 5, including a RAS in 2013, failed to find further evidence of *S. japonica* which suggests that this first colonisation attempt was unsuccessful. It was, however, found in a nearby marina in 2012 (Plymouth 3, Fig. 3b) where it has persisted and spread within a year to an adjacent, newly opened marina (Plymouth 2).

### Distribution in other European sites

A single record of *S. japonica* has been noted from a cruise-ship pontoon in Douglas on the Isle of Man (EJ Cottier-Cook, pers. comm. Fig. 1).

*Schizoporella japonica* was not found in any of the Northern Irish marinas surveyed. In the Republic of Ireland *S. japonica* was found in only one out of the 28 marinas surveyed, fouling buoys on the visitor pontoons of Greystones Marina, to the south of Dublin (Figs. 1, 3a). This represents the first observation of the species in Ireland.

Porter et al. (2015) reported finding *S. japonica* in three out of six Norwegian marinas surveyed in 2014 (Fig. 1).

*Schizoporella japonica* was not found in any of the 13 marinas surveyed in NW France (Bishop et al. 2015a) (Fig. 1) or the 3 marinas surveyed in Portugal (JL unpubl. obs.).

### Substrata

*Schizoporella japonica* has been found attached to a variety of substrata, including: plastic, wood, concrete and metal pontoons; buoys, ropes and chains; and recreational and commercial boat hulls. It was found fouling shellfish (Ryland et al. 2014; Collin et al. 2015), salmon cages in Shetland (JSP pers. obs.), and wave and tidal energy devices in Orkney (Ryland et al. 2014; Nall 2015) and the west coast of Scotland (JL pers. obs.). It has also been found growing epibiotically on organisms attached to man-made surfaces, including solitary ascidians, calcified worm tubes, mussels, barnacles and other encrusting bryozoans; it is often found on kelp holdfasts although rarely on other algae.

In Stromness, Orkney, *S. japonica* was found in August 2014 colonising a large rock in the intertidal zone of the rocky shore line (Fig. 3c). This natural substrate was being used to weigh down a cauldron, a makeshift anchor, to which a small boat chain was attached. This is not the only record of *S. japonica* colonising rocks in the vicinity of boating activities as it was also recorded within Lerwick harbour, Shetland on bedrock and boulders by JSP later in the same month (Collin et al. 2015) (Fig. 3d).

## Discussion and conclusions

*Schizoporella japonica* was introduced to GB in or before 2009 and has since acquired an extensive but widely discontinuous distribution in GB and Ireland. Although frequent in marinas and harbours in Scotland, its few sites in Wales, England and Ireland are separated by wide gaps documented as genuine absences by rapid assessment surveys or targeted searches. This study adds 15 new GB observations of *S. japonica*, 12 of which are in Scotland. The new records in Scotland may represent an expanding range for the species, but could also be a result of a lack of survey effort prior to its initial discovery.

Despite the species first being reported from Wales in 2010 (Ryland et al. 2014) and the 2009 record in England noted here, in Scotland *S. japonica* was found in 28% of surveyed sites, compared to just 6% of surveyed sites in the rest of GB (see Supplementary material for detailed presence and absence records). This is evidence of the species’ ability to persist and spread in the relatively cooler northern waters enabled by its wide temperature tolerance as indicated by its successful introduction to sites with temperatures ranging as low as 4 °C in Northern Scotland (Loxton 2014) and as high as 30 °C in Langkawi, Malaysia (Taylor and Tan 2015; NOAA 2016). Its cold-water tolerance sets the species apart from other non-native bryozoans in Europe, which are predominantly warm-water species e.g. *Bugula* and *Bugulina* spp. (Ryland et al. 2011).

The initial records of *S. japonica* in GB were the first for the Atlantic Ocean, and thus appear to represent primary introduction from a different biogeographical region. Most other recent arrivals of non-native sessile invertebrates to GB seem to have come as secondary introductions from Europe (often apparently moving northwards across the English Channel from France) (Bishop et al. 2015a); their subsequent spread within GB has thus been predominantly northward. Similarly, introduced algae have generally reached GB as secondary introductions from France, with commercial imports of oysters to France being the predominant vector of primary introduction to Europe (e.g. Verlaque 2001). This has all contributed to the pattern whereby the first occurrences of the majority of marine non-native species in GB have been on the English Channel coast (Minchin et al. 2013).

In the NW Atlantic, *S. japonica* has been reported up to 63°N in Norway and as far south as Plymouth at 50°N where, despite its apparent failure to establish in one marina, the subsequent colonization of two other sites indicate that the conditions are generally suitable. The species’ occurrence as reproductive colonies in Malaysia (Taylor and Tan 2015) indicates that its tolerance of higher temperatures elsewhere in the world would, in the Atlantic, allow substantial southward extension of its current range, potentially into tropical waters. However, the species has not yet been found on the opposite side of the English Channel in NW France, or further south in Portugal.

The current GB distribution of *S. japonica* raises the possibility of relatively long-term unreported presence in Scotland prior to its discovery there, possibly due to haphazard survey effort and the absence of the species from horizon-scan lists. This would have enabled the relatively high proportion of sites hosting *S. japonica* now observed in Scotland to build up. It seems likely that *S. japonica* is a rare example of a southward-spreading species in GB and that we are now observing saltatory secondary spread from a Scottish bridgehead by anthropogenic vector(s), with the expectation of back-filling of distribution gaps over time. According to this scenario, the recently determined record of the species from the extreme SW of GB in 2009 would not pre-date the actual arrival of *S. japonica* in Scotland.

Currently the reasons for the species’ discontinuous distribution in GB and Ireland can only be hypothesised, but continued monitoring to plot the pattern of ongoing spread may enable better inference of the species’ history and identify the vectors responsible. Niche modelling to more accurately predict future movements is also recommended. Genetic analysis of both native and non-native populations would also be useful in documenting the species’ phylogeographic history, verifying potential vectors, and elucidating any cryptic speciation or geographical clades.

As a result of *S. japonica*’s short larval dispersal duration (Treibergs 2012), this species is unlikely to have been able to spread around GB as fast as it has by natural means alone. Human vectors such as recreational boating, commercial vessel movements, and aquaculture stock and equipment movement are therefore likely to have contributed to its seemingly rapid spread and wide-reaching but discontinuous distribution, as they have for many other marine bioinvasions (Keller et al. 2011; Minchin et al. 2013). The prevalence of *S. japonica* in the shallow subtidal area of floating structures (pontoons and buoys) makes vessel hull fouling a particularly likely vector for this species because boats are analogous to these structures. Vessel hull fouling has previously been implicated in *S. japonica* introductions elsewhere in the world (Ashton et al. 2014) and it may well be the primary vector for its spread around GB and Ireland. In fact, the Royal Yachting Association reports heavy use of sailing routes between a number of sites where *S. japonica* is present, such as the one between Holyhead, the Isle of Man and the marinas around Dublin. We expect *S. japonica* to spread in future around suitable sections of the English, Welsh and Irish coasts, and further within Europe, via vessel hull fouling of both recreational and commercial vessels.

Although we note in this paper the relative novelty of a cold-tolerant species arriving in Northern Europe and spreading South, in the future this may become more commonplace, making *S. japonica* a bellwether of future invasion patterns. As our climate warms and sea ice continues to reduce year on year (Rhein et al. 2013), the opening of new Arctic trade routes may affect the distribution of invasive species (Miller and Ruiz 2014), especially for cold-tolerant species like *S. japonica*. The Northwest passage and the Northern Sea Route could allow cold-tolerant hull fouling species to move to Europe from North America to the West and from Russia and East Asian ports to the East; once arrived in Europe, species with wide temperature tolerance ranges, like we have seen with *S. japonica,* may be expected to establish reproducing populations. There is also a risk of the introduction of European species into new locations such as the high-Arctic archipelago of Svalbard.

The development of the marine renewable energy industry may also contribute to the species future spread. Marine renewable devices provide an ideal habitat for *S. japonica* as many are floating (thus offering shallow yet permanently submerged surfaces) and are predominantly not coated in antifouling paints (Nall 2015). These devices will also be stored and maintained in harbours known to contain *S. japonica* (Nall et al. 2015) which provides an ideal opportunity for colonisation and then subsequent spread when the device is wet-towed to the energy extraction site or to another harbour (Nall 2015).

The occurrence of *S. japonica* fouling a wide variety of artificial substrates associated with commercial activity highlights the economic threat posed to marine industries by its ongoing spread. A particular industry at risk is the mussel and oyster industry, where biofouling can render underwater gear and lines cumbersome and can heighten competition for food and even smother mussels and oysters. Growth on mussels and oysters reduces their commercial value. *S. japonica* also appears capable of rapidly occupying newly available substrate space, as seen during eradication attempts to clear the invasive ascidian *Didemnum vexillum* in Holyhead Marina (Ryland et al. 2014), and at the new marina Plymouth 2, where the species maintained high levels of space occupancy 18 months after heavy colonization of the new pontoons (CAW and JDDB unpubl. obs.).

Although this species is typically associated with human activity, it has been observed in the natural environment fouling rocks and boulders in both Orkney and Shetland. *S. japonica* has become widespread intertidally on the West coast of North America on rocks, boulders and shellfish since its introduction in 1938 (Dick et al. 2005). In Alaska, *S. japonica* has been shown to out-compete native encrusting bryozoan species (Dick et al. 2005). Observations of *S. japonica* on rocks and boulders in Scotland may, therefore, represent a risk of the species becoming more widespread in the intertidal zones of GB and Ireland over time. *S. japonica* is a strong competitor for space and is known to inhibit growth of adjacent species and overgrow some native species, such as other bryozoans and the blue mussel *Mytilus edulis*, in some cases causing mortality (Treibergs 2012; Macleod et al. 2016). *S. japonica* appears able to proliferate in cold water conditions, thereby taking advantage of conditions when native species may be dormant (Ryland et al. 2014). Given its competitive ability, the introduction of this species could, therefore, also have a negative impact on native biodiversity, including in UK priority habitats such as intertidal under boulder communities and mussel beds (Macleod et al. 2016).

Non-native species that are capable of rapid spread, either naturally or aided by anthropogenic vectors, and can have negative impacts on native ecosystems and on economic activity are termed “invasive” (Keller et al. 2011). As a result of its high abundance and dominance in harbours, Nall et al. (2015) reported that *S. japonica* was already showing signs of being invasive in the UK and the findings of this paper support this view. The authors suggest that *S. japonica* should now be considered invasive in GB and Ireland. As such, it is recommended that biosecurity procedures alongside effective surveillance and monitoring should be prioritised for regions outside the species’ current distribution, in keeping with the species’ inclusion on the UK’s priority monitoring list for MSFD Descriptor 2 of Good Environmental Status.

## Electronic supplementary material

Below is the link to the electronic supplementary material.

Supplementary material 1 (XLSX 52 kb)
